# Protective Effect of Autologous Arteriovenous Fistulae Against Oxidative Stress in Hemodialyzed Patients

**DOI:** 10.7759/cureus.15398

**Published:** 2021-06-02

**Authors:** Rodolfo A Valtuille, Guillermo Rossi, Eliana Gimenez

**Affiliations:** 1 Dialysis Unit, Fresenius Medical Care, Burzaco, ARG; 2 Clinical Biochemistry, Laboratorio Rossi, Adrogué, ARG

**Keywords:** oxidative stress, chronic kidney disease, hemodialysis, vascular access, inflammation

## Abstract

Introduction: Oxidative stress (OS) is ubiquitous in chronic kidney disease (CKD) and is exacerbated by hemodialysis (HD). OS is also associated with anemia, malnutrition, and cardiovascular (CV) disease and is an independent predictor of mortality and morbidity in patients undergoing HD. HD vascular access (VA) types are strongly correlated with CKD patient outcomes. Prolonged use of central venous catheters (CVC) for HD and arteriovenous grafts (AVG) promotes inflammation and OS. However, the effects of the VA type on OS have been poorly studied in HD patients. This study investigated OS prevalence in an HD population to determine the relationship between the VA type and HD.

Methods: The oxidative stress index (OSI) was used to assess the HD patients’ OS status. OSI summarizes information derived from the reactive oxygen metabolites (d-ROMs) fast test and the plasma antioxidant test (PAT) in a single value, using the hydrogen peroxide concentration (for d-ROMs) and ascorbic acid (for PAT) as reference standards. The OSI was created to indicate how far the OS status deviates from normal (i.e., fully compensated oxidative balance). An index increase may be from an increase or decrease in peroxide or antioxidant concentrations. Patients undergoing chronic HD were evaluated by dividing the cases according to the OSI status: normal (N-OSI), borderline (BL-OSI), high (H-OSI), and very high (VH-OSI). Patients with clinical evidence of active infections were excluded.

Results: In total, 129 patients were included; 86.8% used high-flux dialyzers, 13.2% used hemodiafiltration (HDF), and 24.5% were diabetic. An altered OSI was observed in 86 of 129 patients (66.7%). An increased OSI correlated with a significant increase in d-ROMs (r = 0.420) and PAT (r = 0.710). There were no differences between sex, diabetes status, age, dialysis vintage, or dialysis modalities. d-ROMs were inversely correlated with hemoglobin levels (r = −0.209). The iron dose by month correlated with the OSI (r = 0.189) and was significantly lower in the N-OSI group. N- and BL-OSI patients had a significantly higher rate of autologous arteriovenous fistula (AVF) compared to the other groups, and VH-OSI patients had a higher rate of permanent tunneled CVC.

Conclusion: Most HD patients had more OS, indicated by the OSI scores. In chronic HD patients, AVF had a protective effect against imbalanced peroxidation-antioxidation.

## Introduction

Oxidative stress (OS) is an imbalance between free radical (i.e., reactive oxygen species, ROS) production and the existing antioxidant capacity (AC) [1]. OS is implicated in various pathologic pathways, such as diabetes mellitus, atherosclerosis, inflammation, and chronic kidney disease (CKD) [2,3]. OS is ubiquitous in CKD and exacerbated by hemodialysis (HD) [2,3]. OS in HD is associated with renal anemia, malnutrition, and cardiovascular (CV) disease and has been reported as an independent predictor of mortality and morbidity for HD patients [4,5].

In HD, dialysis membrane biocompatibility, dialysate bacteriologic quality, parenteral iron administration, and the vascular access (VA) type potentially aggravate OS [2,3]. Specifically, the VA type has been strongly correlated with HD patient outcomes [6]. Therefore, it was speculated that the prolonged use of central venous catheters (CVC), malfunctioning arteriovenous grafts (AVG), and arteriovenous fistulae (AVF) promoted inflammation and OS [7]. A study that included 11 patients with a native AVF and 15 patients with an AVG showed elevated OS biomarkers in intima and neointima samples recovered after surgery in all cases, even in VAs without clinical signs of infection [8]. However, the effect of VAs on OS is unclear in HD patients.

There are three suggested approaches to assess OS in the clinical setting: directly measuring ROS levels, indirectly measuring ROS levels based on the detection of oxidative damage byproducts (e.g., DNA, lipids, and proteins), and measuring the AC (e.g., enzymatic antioxidant activities, non-enzymatic antioxidant activities, or total AC) [1]. The oxidative stress index (OSI) was developed to summarize the information derived from the reactive oxygen metabolites (d-ROMs) fast test and the plasma antioxidant test (PAT) in a single value [9]. Hydrogen peroxide (for d-ROMs) and ascorbic acid (for PAT; i.e., vitamin C) concentrations are used as OS and AC reference standards, respectively. The OSI was developed to indicate how far OS deviates from normal (i.e., the full compensation of oxidative balance); as the OS deviation increases, the OSI score increases. Using this method, when the d-ROMs and PAT results are within a normal range, the OSI score will also be within a normal range (<40). Increases in the index score could be the result of either increases or decreases in peroxide or antioxidant concentrations. This study used the OSI to analyze the prevalence of OS in HD patients and determine how the VA type affects OS status.

This article was previously presented as a meeting poster at the 56th ERA-EDTA Congress on June 14, 2019.

## Materials and methods

In total, 129 patients undergoing chronic renal replacement treatment were included in the study; 86.8% used high-flux dialyzers containing a polysulfone membrane, and 13.2% used on-line hemodiafiltration (HDF) with a Helixone membrane. Patients with clinical evidence of active infection or a recent hospitalization (less than 30 days prior) were excluded. All patients signed informed consent forms at admission to the institution.

Renal replacement therapy was administered in thrice-weekly, four-hour sessions, using bicarbonate-buffered dialysate. Anticoagulation during dialysis was performed with unfractionated heparin. Parenteral iron sucrose injection was used to correct iron deficiencies. Obstructed AVGs with or without signs of infections were routinely removed. Blood samples for OSI, d-ROMs, and PAT were drawn from the VA simultaneously with the routine monthly laboratory tests before starting the dialysis procedure and at the mid-week HD session. Parenteral iron supplementation was suspended during the sampling week. Bacteriological water quality was surveyed monthly with water cultures and Limulus-amoebocyte-lysate tests for endotoxins.

The OSI was scored using a free radical analytical system (FRAS 5; H&D SRL, Pharma, Italy) [9] to assess the OS status in HD patients. For d-ROMs, the reference range was between 250 and 300 Carratelli Units (Carr. U.). For PAT, values >2800 Cornelli Units (Cor. U.) were considered standard, and values <1800 Cor. U. were considered deficient.

Data were analyzed using MedCalc package version 19.5.3 for Statistical Analysis (MedCalc Software, Ltd., Ostend, Belgium) [10]. The population was divided according to the OSI status as follows: normal (N-OSI; <40), borderline (BL-OSI; 41-65), high (H-OSI; 66-120), and very high (VH-OSI; >120). Comparisons between groups were performed using a one-way analysis of variance. Correlation coefficients were determined using the Pearson method. All values are presented as the mean ± standard deviation (SD) or median (range) as appropriate. A p-value <0.05 was statistically significant.

## Results

Patient characteristics and OS-related variables are presented in Table 1. An altered OSI (i.e., the BL-, H-, or VH-OSI groups) was observed in 86 of 129 patients (66.7 %; Table 2). An increased OSI score correlated with a significant increase in d-ROMs (r = 0.420; p < 0.0001) and PAT (r = 0.710; p < 0.0001; Table 2) scores. OSI did not correlate with age or dialysis vintage (Tables 3 and 4) but was correlated with the iron dose by month (r = 0.189; p = 0.0345; Table 3), which was significantly lower in the N-OSI group (Table 4). d-ROMs inversely correlated with the hemoglobin (Hb) level (r = −0.209; p = 0.0188; Table 3).

**Table 1:** Patient characteristics, OS-related biomarkers, and routinely measured variables. OS: oxidative stress; SD: standard deviation; 25–75 P: 25th-75‑percentiles; U/BW: unit/body weight; OSI: oxidative stress index; Carr. U.: Carratelli units; Cor. U.: Cornelli units.

	Mean	SD	Median	25–75 P
Age (years)	60	15	63	51–71
Vintage (months)	55	64	30	11–66
Albumin (g/dL)	3.89	0.34	3.9	3.7–4.1
Hemoglobin (g/dL)	11.4	1.41	11.4	10.6–12.2
Ferritin (μg/L)	889	514	829	475–1297
Iron dose (mg/month)	216	146	200	100–400
EPO (U/BW)	54	48	39	22–88
Kt/V	1.29	0.21	1.25	1.2–1.4
OSI	84.8	75.6	60	34–113
d-ROMs (Carr. U.)	346.6	120.7	331	257–413
PAT (Cor. U.)	2875.4	1058.3	2641	2415–2908
Bodyweight (kg)	72	16	71	59–84

**Table 2:** OS-related biomarkers [mean (SD)] and VA (%) prevalence based on the OSI status. Statistical significance level, p <0.05. OS: oxidative stress; SD: standard deviation); VA: vascular access; OSI: oxidative stress index; Carr. U.: Carratelli Units; Cor. U.: Cornelli Units; AVF: arteriovenous fistulae; PTFE: polytetrafluoroethylene prosthetic graft vascular access; PERM: permanent catheter; TRANSIT: transient catheter. OSI, d-ROMs, and PAT comparisons are by one-way analysis of variance. Percentage comparisons indicate p-values for trending purposes.

Patients (n, 129)	Normal (N, 43)	Borderline (31)	High (28)	Very high (27)	p-Value
OSI	26 (9)	55 (6)	91 (17)	205 (75)	<0.001
d-ROMs (Carr. U.)	275 (98)	298 (94)	539 (104)	465 (161)	<0.001
PAT (Cor. U.)	2645 (248)	2575 (369)	2653 (539)	3815 (1947)	<0.001
AVF (%)	41.8	26.4	19.8	12.1	0.0006
PTFE (%)	21.4	21.4	28.6	28.6	0.96
PERM (%)	6.2	18.8	12.5	62.5	0.0006
TRANSIT (%)	12.5	12.5	50	25	0.39

**Table 3:** Correlogram between OS-related biomarkers and routinely measured parameters. Significance level, p < 0.05. OS: oxidative stress; OSI: oxidative stress index; Carr. U.: Carratelli Units; Cor. U.: Cornelli Units; BW: body weight.

	OSI	d-ROMs (Carr. U.)	PAT (Cor. U.)	Vintage (months)	Age (years)	Albumin (g/dL)	EPO (kg/BW)	Iron (mg/month)	Ferritin (μg/L)	Hb (g/dL)
OSI p		0.420 (<0.0001)	0.710 (<0.0001)	−0.109 (0.2170)	0.132 (0.1349)	−0.130 (0.1434)	0.116 (0.1971)	0.189 (0.0345)	−0.084 (0.3510)	−0.155 (0.0831)
d-ROMs p	0.420 (<0.0001)		−0.076 (0.3897)	−0.041 (0.6460)	−0.010 (0.9097)	−0.159 (0.0732)	0.110 (0.2236)	0.161 (0.0722)	−0.131 (0.1444)	−0.209 (0.0188)
PAT p	0.710 (<0.0001)	−0.076 (0.3897)		−0.122 (0.1671)	0.107 (0.2286)	−0.065 (0.4654)	0.091 (0.3106)	0.084 (0.3522)	−0.050 (0.5786)	−0.079 (0.3783)

**Table 4:** Routinely measured biomarkers and patient parameters based on the OSI status. The values expressed as the mean (SD). Statistical significance level, p<0.05. OSI: oxidative stress index; Hb: hemoglobin; EPO: erythropoietin; U/BW: units/body weight.

Patients (n, 129)	Normal (N, 43)	Borderline (31)	High (28)	Very high (27)	p-Value
Hb (g/dL)	11.6 (1.4)	11.6 (1.4)	11 (1.5)	11 (1.1)	0.12
Albumin (g/dL)	3.9 (0.3)	3.9 (0.4)	3.8 (0.2)	3.8 (0.3)	0.54
Ferritin (µg/L)	1003 (535)	786 (452)	933 (520)	784 (522)	0.20
EPO dose (U/BW)	54 (54)	40 (39)	62 (49)	64 (46)	0.28
Iron (mg/month)	157 (139)	254 (131)	229 (148)	256 (147)	N vs. BL *p*<0.05
Age (years)	56 (16)	63 (13)	62 (14)	62 (17)	0.15
Vintage (months)	62 (65)	56 (65)	59 (62)	37 (41)	0.45

There were no differences in the mean OSI scores between sexes (female = 92 versus male = 80; p = 0.9), based on diabetes status (non-diabetics = 87 versus diabetics = 72; p = 0.16), or based on dialysis modalities (HD = 87 versus HDF = 77; p = 0.9).

**Figure 1:**
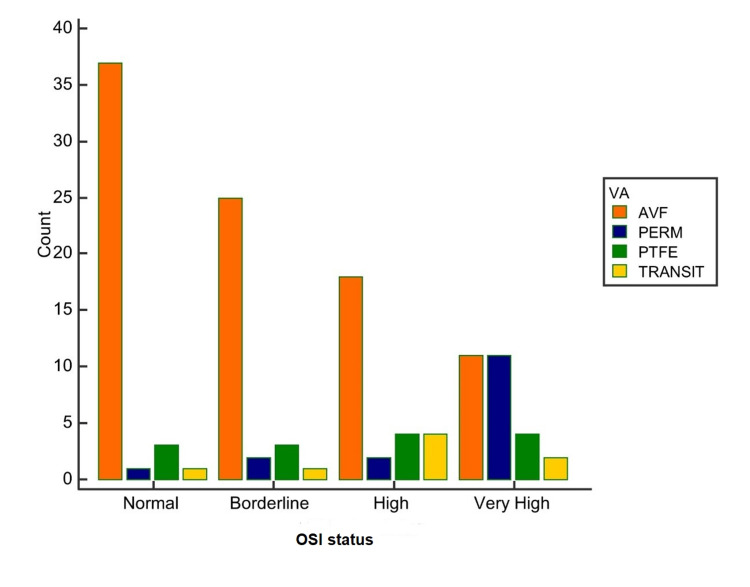
Vascular access distribution based on OSI. OSI: oxidative stress index; VA: vascular access; AVF: arteriovenous fistulae; PTFE: polytetrafluoroethylene prosthetic graft vascular access; PERM: permanent catheter; TRANSIT: transient catheter.

Regarding VA types, 70.5% of patients had an autologous AVF, 12.4% had a tunneled permanent catheter (PERM), 10.9% had a polytetrafluoroethylene (PTFE) AVG, and 6.2% had a transient catheter (Table 5 and Figure 1).

**Table 5:** Cross-tabulation between OSI (columns) and vascular access (rows) categories. The table cells contain the number of times a particular category combination occurred. The table margins contain the total number of observations per category. OSI: oxidative index status; VA: vascular access; AVF: arteriovenous fistulae; RT: % of row total; CT: % of column total; PERM: permanent catheter; PTFE: polytetrafluoroethylene prosthetic graft vascular access; TRANSIT: transient catheter.

		OSI			
VA	Normal	Borderline	High	Very high	
AVF	38	24	18	11	91 (70.5%)
RT	41.8%	26.4%	19.8%	12.1%	
CT	88.4%	77.4%	64.3%	40.7%	
PERM	1	3	2	10	16 (12.4%)
RT	6.2%	18.8%	12.5%	62.5%	
CT	2.3%	9.7%	7.1%	37%	
PTFE	3	3	4	4	14 (10.9%)
RT	21.4%	21.4%	28.6%	28.6%	
CT	7%	9.7%	14.3%	14.8%	
TRANSIT	1	1	4	2	8 (6.2%)
RT	12.5%	12.5%	50%	25%	
CT	2.3%	3.2%	14.3%	7.4%	
	43	31	28	27	129
	33.3%	24%	21.7%	20.9%	

Based on the OSI status, patients in the N- or BL-OSI groups showed a significantly higher autologous AVF rate (41.8% and 26.4%, respectively) compared to the other groups. The VH-OSI group correlated with an increase in the PERM central catheter rate (Tables 2 and 5 and Figure 1).

## Discussion

In this cross-sectional study, 66.7% of chronic HD patients had an altered OSI. It is known that OS increases in the later stages of CKD and becomes more severe in HD patients [2,3]. However, the true prevalence of increased OS is difficult to determine because of the numerous approaches to measuring OS [1-3]. For example, a study using only d-ROMs showed abnormally higher values (>300 Carr. U.). in nearly 50% of cases (258 of 517) [5].

OSI considers the oxidizing and antioxidant components in a single calculation for a more integrated approach, rather than using only isolated markers (e.g., enzyme activity and lipid degradation products); the calculation acknowledges that OS is a dynamic process [1]. In our study, the OSI value derived from d-ROMs and PAT represents OS and AC using an automated commercial approach [9]. Moreover, the index value will only be within a normal range when both tests are within their normal ranges. The use of the term OSI in the literature is broad, and it does not only include the relationship between d-ROMs and PAT [1]. Therefore, it is essential to know which components are being represented under the term OSI.

Some studies used biological antioxidant potential (BAP) instead of PAT to measure AC in HD patients [11,12]. Iron reduction is the basis for the BAP and PAT methods. However, zirconium salts in PAT avoid phosphate interference, which is crucial for HD patients [13]. Our study is the first to use OSI with d-ROMs and PAT in HD patients, but several publications used similar approaches with only one component (d-ROMs or PAT/BAP) and reported altered OS in CKD patients [5,11,12,14,15]. In three studies [11,12,15], d-ROMs were higher in CKD patients compared with non-CKD patients. Further, d-ROMs inversely correlated with the estimated glomerular filtration rate and proteinuria [11] and acted as a predictor of CV events [15].

A critical study by Sasaki et al. examined 517 HD patients for five years, and d-ROMs positively correlated with the C-reactive protein (CRP) level [5]. Further, their Kaplan-Meier analyses showed that higher d-ROMs and CRP levels predicted a higher risk of CV events and mortality [5]. These results are comparable to another cohort study that used myeloperoxidase as a surrogate and reported that OS predicted all-cause mortality [4]. Unfortunately, CRP is not routinely measured in our institution. Thus, we cannot make result comparisons.

In our study, an increased OSI correlated with a significant increase in d-ROMs and PAT, which were previously correlated with CKD patient outcomes with or without HD [11,12,14,15]. Several dialysis-related factors aggravate the multifactorial proinflammatory and pro-oxidative CKD status [2,3]. Non-compatible membranes and dialysates with a high bacterial count during HD promote the formation and accumulation of oxidative products by activating platelets, complement, and polymorphonuclear white blood cells [2,3,7]. In our study, some of these factors likely did not cause the altered OS status because compatible membranes (helixone and polysulphone) were used and the water quality was routinely monitored. HDF uses ultrapure water and can clear prooxidants better than standard HD, which has a positive effect on OS [16]. The OS status did not differ between the HD and HDF groups, but this may be attributed to the lower number of HDF patients.

A patient’s anemia status and the drugs used to correct anemia, especially parenteral iron, also correlate with worsening OS in HD patients [2,3]. Erythropoietin (EPO), used to treat anemia, significantly inhibited the oxidation process, suggesting that anemia aggravates OS [2,3]. In our experience, OSI was independent of the EPO dose per month, but d-ROMs inversely correlated with the Hb level. Co-adjuvant treatment of anemia with intravenous (IV) iron administration was linked with an oxidative response in HD patients. Its deleterious effect was related to the dose and the time of infusion [3]. We found an association between the iron dose and OS, even though iron was slowly delivered during HD [2,3] and administration was stopped one week before blood samples were taken. Adding iron to the dialysate (ferric pyrophosphate citrate) is a new approach to avoiding OS triggered by IV administration [17]. Iron from dialysate seems to be quickly removed from circulation during HD, which is useful for preventing its pro-oxidative effect [18].

Native AVF is the preferred VA type to ensure dialysis adequacy and improved patient outcomes [6]. Using CVC for VA and malfunctioning AVG are known to promote OS-inflammation [7], but the effect of the VA type on OS has been inadequately analyzed in HD patients. The most critical finding of this study was the overwhelming effect of the VA type on the OSI status. The VA prevalence analysis based on the OSI status showed that N-OSI patients had a significantly higher autologous AVF rate (AVF: 37/42, 88%) compared to the other groups. Conversely, VH-OSI patients had an increased CVC and decreased AVF rate (AVF: 11/28, 39%; Table 5) compared to the other groups.

Our results agree with those of Weiss et al. [8], who showed increased OS markers, neointimal proliferation, and inflammation in autologous AVF and PTFE AVG at the time of surgical revision or resection. Conversely, our results reinforced the CHOICE (Choices for Healthy Outcomes in Caring for end-stage renal disease) study findings [19], which showed that CVC was associated with a significant state of inflammation (e.g., elevated CRP and interleukin 6) and higher mortality, supporting the classic recommendations and the current consensus regarding AVF as the first VA choice for HD [20,21].

Some limitations of this study should be mentioned. This was a single-center study using OSI for the first time in HD patients, and our results are the first to demonstrate a strong relationship between the VA type and OS in this patient population. In our experience, an altered OS status could be predicted based on the VA type and the presence of an autologous AVF, which had a protective effect. The effects of inflammation (infectious or non-infectious) and the VA duration on OS remain unresolved. Further, this study lacked a designed control group.

## Conclusions

HD patients had a high prevalence of OS, indicated by the OSI scores. However, autologous AVFs had a protective effect against imbalance peroxidation-antioxidation. A new controlled multicenter prospective study evaluating the effects of inflammation (infectious or non-infectious), the VA duration as well as the replacement of a VA for another OS, could reinforce these findings.
